# Sport events’ impact and legacy on quality of life: a scoping review

**DOI:** 10.3389/fspor.2025.1681463

**Published:** 2025-10-30

**Authors:** Haohan Xu, Shintaro Sato

**Affiliations:** ^1^ Graduate school of Sport Sciences, Waseda University, Tokyo, Japan; ^2^ Faculty of Sport Sciences, Waseda University, Tokyo, Japan

**Keywords:** subjective well-being, quality of life, sport events, impact, legacy

## Abstract

**Research purpose:**

The purpose of this research was to map and evaluate the existing literature on the impacts and legacies of sport events on quality of life outcomes, identifying trends, gaps and proposing future research directions. Based on the analysis of 70 studies that examines sport events’ impact and legacies on quality of life, this scoping review aims to address three key questions: (1) What is the current state of the literature in terms of studied context and research design? (2) Which theoretical frameworks underpin the studies? (3) What are the key gaps in this research domain?

**Research method:**

The scoping review follows the five-stage framework proposed by Arksey and O'Malley.

**Results and findings:**

(1) Predominant focus on mega/large events, heavy reliance on subjective measurements; (2) frequent absence of theoretical frameworks (3) Limited understanding of the distinct psychological mechanisms across event types that underlie quality of life outcomes.

## Introduction

Sport events have become increasingly popular and influential in today's society, the power of sport events to bring people together, create a sense of community, and promote physical and psychological well-being has been widely recognized (2–4). In recent years, academic interest in examining the multifaceted impacts and legacies of sport events has grown significantly, largely because sport events are capable of generating broad spectrum of impacts that are closely tied to individuals' holistic evaluations of well-being, commonly referred to as quality of life (2, 5, 6).

However, the literature on the impact and legacies of sport events on quality of life revealed mixed findings. While their multidimensional nature (economic, socio-cultural, environmental) is consistently acknowledged (7–9), studies conflict on outcomes. Positive effects like enhanced community pride, international image, and environmental awareness (10, 11) are linked to quality of life perceptions (4, 12, 13), yet others highlight negligible or adverse impacts, such as rising living costs, disruption of everyday life (14–16). These inconsistency stem from variations in measurement timing; divergent approaches to assessing quality of life across general vs. specific populations; and substantial differences in sample size and participant characteristics (17–19). Consequently, they underscore the need for a scoping review to systematically map existing evidence, clarify conceptual and methodological gaps, and explore the contextual factors shaping outcomes.

This scoping review will achieve this by addressing the following three areas. First, the current study will identify patterns, trends, and gaps in the contexts and research designs studied within the current literature. Second, it will analyze the theoretical frameworks applied in existing studies. Third, we will pinpoint gaps and propose directions for future investigations.

To achieve the above, we will employ a statistical characteristics analysis, utilizing extracted data to address the research context, methods, design and measurements in the studies. By presenting the current state of literature on how sport events relate to quality of life, this review offers a clear roadmap for scholars to refine theories, adopt more rigorous methods, and propose novel lines of inquiry, ultimately advancing the study of sport events and their relationship to quality of life.

As society continuously seeks to thrive and achieve greater quality of life outcomes (20, 21), it becomes crucial to explore the distinctive contributions of sport events to these aspirations and their role in promoting overall well-being and quality of life within the population (6, 8). Understanding the mechanisms by which sport events impact quality of life can enable us to more effectively maximize the events' potential to foster societal happiness and individual quality of life (22).

## Literature review

### Event impact and legacy

Impact and legacy are two different constructs. Legacy refers to the longer-lasting alterations in a host city's infrastructure, knowledge base, or networks, which continue to influence outcomes well beyond the event's conclusion (6). Impact primarily refers to the short-term impulse caused by the event, such as event-related visitor spending or temporal changes occurred (6). In previous sport event impact studies, impact has been defined as the perceived positive or negative changes that occurred or will be manifested due to hosting of such events (23–25).

The concept of legacy is defined as both planned and unplanned, positive, and negative, tangible, and intangible outcomes that persist after the event (6). This definition of legacy has been widely applied in research examining sport event's outcomes (26, 27).

However, in the examination of sport events and their relationship to society, the terms “impacts” and “legacies” are often used interchangeably (28). The scholarly exploration of sport events' impact and legacy has experienced a significant evolution, transitioning from an initial focus on economic outcomes to a more comprehensive understanding of their multifaceted impacts (29), encompassing economic, socio-cultural, and environmental dimensions (3, 4). Previously in the late 20th century, legacy studies predominantly centered on mega-events like the Olympic Games, with an emphasis on tangible structures, such as job creation, revenue generation, and/or infrastructure-related projects remaining post-event (6, 30–32).

Whereas scholars still give keen attention to positive economic impact derived from sport events, a paradigm shift occurred, driven by growing skepticism about the overestimation of economic impact (33) and an increasing acknowledgment of potential negative economic consequences, such as cost overruns and underutilized infrastructure (34). The Sydney Olympics in 2000 and London Olympics in 2012 marked a pivotal moment in changing the narrative around sport events (35). As the International Olympic Committee (IOC) integrated legacy planning into its bid requirements, emphasizing the importance of lasting positive impacts on host cities and residents, thus fueling academic discussion and research into various dimensions of legacy (36, 37).

### Quality of life & subjective well-being

Scholars have shown keen interests in how hosting events can shape local communities' quality of life and well-being (18, 38, 39). This pursuit aligns closely with key concepts in positive psychology (40). One of the key concepts is quality of life, as defined by the World Health Organization (41), encompasses individuals' perceptions of their position in life within their cultural and value systems. This perception is influenced by factors such as physical health, psychological state, level of independence, social relationships, and environmental features. In mainstream psychology, Pavot and Diener (42) similarly characterize subjective quality of life as a person's conscious evaluative judgment of their own life, guided by personally determined standards. Quality of life, therefore, is a multidimensional construct encompassing a range of individual life aspects and their environmental interplay (43).

In this review, quality of life is defined as subjective assessment of an individual's state of being (34), articles utilizing this working definition will be considered for this review. Subjective well-being, a proximal construct within this theoretical domain, articles are included if subjective well-being is defined as, evaluation of individuals' perceptions of their own quality of life, including their affective states, overall life satisfaction, and evaluations of specific life domains (44). Research that utilizes life satisfaction is considered eligible if is operationalized as attitude that arises from a global cognitive evaluation of one's satisfaction with life (45).

As these constructs emerge from the broader field of positive psychology, researchers have often used quality of life, subjective well-being and life satisfaction interchangeably as measures of residents' holistic life evaluations in studies examining the impact of sport events on host communities (15, 27). Although quality of life is multifaceted, encompassing psychological, social outcomes, it orients around the measurement of one's subjective experience of life or subjective well-being (34). This is also evident in the literature through the use of single-item measures of overall life satisfaction (46).

## Methods

The methods for this scoping review follows the five-stage framework proposed by Arksey and O'Malley (1), which has been enhanced by a data reporting system in alignment with the PRISMA-ScR guidelines. This approach ensures a thorough exploration of the existing academic contributions while remaining adaptable to the vast nature of the available research.

This scoping review was designed to provide a comprehensive overview of the research landscape concerning the impacts and legacies of sport events for quality of life outcomes. By elucidating the volume of literature available, the diversity of studies conducted, and identifying both methods and knowledge gaps, this review adopts a panoramic approach as outlined by Grant and Booth (47).

Unlike other systematic review and meta-analysis projects, this scoping review did not perform a critical appraisal or synthesize findings from the included studies. Instead, it allowed for the inclusion of a wide array of relevant literature without stringent evaluative criteria, focusing on understanding the breadth of the field rather than assessing the efficacy of sport events' impacts on quality of life.

### Research questions

According to Arksey and O'Malley (1), research questions should be designed to guide the search strategy effectively, ensuring broad coverage and the inclusion of all relevant literature while avoiding excessive narrowness that could limit comprehensive analysis. In alignment with the purposes and the objectives of this scoping review, this research seeks to address the following questions, (1) What is the current state of literature that addresses impacts, legacies of sport events on quality of life outcomes in terms of study context and research designs? (2) Which theoretical frameworks underpin the studies in this field? (3) What key gaps and future directions should guide advancements in this domain?

### Search

For comprehensive search of the relevant articles in sport events and quality of life, a combination of electronic database searching and manual searching was employed from anytime to December 31, 2023. The initial step implemented in this study was an electronic database search, employing a series of keywords derived from prior readings (2, 29, 34).

The subsequent search string, “legac* OR impact* OR outcome*” AND quality of life OR well-being OR livelihood OR satisfaction AND “Sport* Event*”, was meticulously developed through close consultation with experts in the field. This process involved reviewing key literature and engaging in discussions with experts to identify the most pertinent and widely used terminology in the discipline.

This search used three major databases: Scopus, Web of Science, and SPORTDiscus. These databases were deemed appropriate as they provided extensive coverage of scholarly literature across various disciplines, including sports science, public health, sociology, and psychology. Scopus and Web of Science were chosen for their broad, multidisciplinary coverage to ensure comprehensive data capture, while SPORTDiscus, a database with a specialized focus on sports and sports-related subjects, was used to identify sport-specific insights often overlooked in general databases.

Search results were exported to Mendeley Reference Manager for analysis, with search terms tailored to each database. A complementary manual search was conducted post initial screening, examined citations/references in selected studies to identify additional sources not captured electronically, ensuring comprehensive coverage. The process adhered to PRISMA-ScR guidelines.

### Eligibility criteria

During the initial phase of the search across online databases, based on the established search string total of 796 relevant studies were identified. These studies were sourced from three different databases: 325 from Web of Science (WoS), 278 from Scopus (Searched within Article Title, Keyword and Abstract), and 193 from SPORTDiscus. The eligibility criteria for inclusion in this study were as follows: the articles had to be peer-reviewed journal articles, written in English, and we included studies from 2007 onward, following Preuss's seminal work establishing holistic legacy frameworks. Furthermore, the articles selected for inclusion needed to be directly relevant to the fields of sport events, in align with legacy defined as the lasting changes, planned or unplanned, tangible and intangible that endure after the event's conclusion (6) and impacts defined as, the positive or negative changes that occurred or will be manifested due to hosting of such events (23).

After applying the eligibility criteria, 665 articles were identified. Following the removal of 222 duplicates, 443 articles remained for screening. All were compiled into a Microsoft Excel file for analysis. A two-stage screening was conducted. The first round reviewed titles, keywords, and abstracts, excluding studies unrelated to quality of life, subjective or objective well-being, and those addressing sports broadly rather than specific sport events, in line with the review's focus on sport event impacts and legacies.

**Figure 1:**
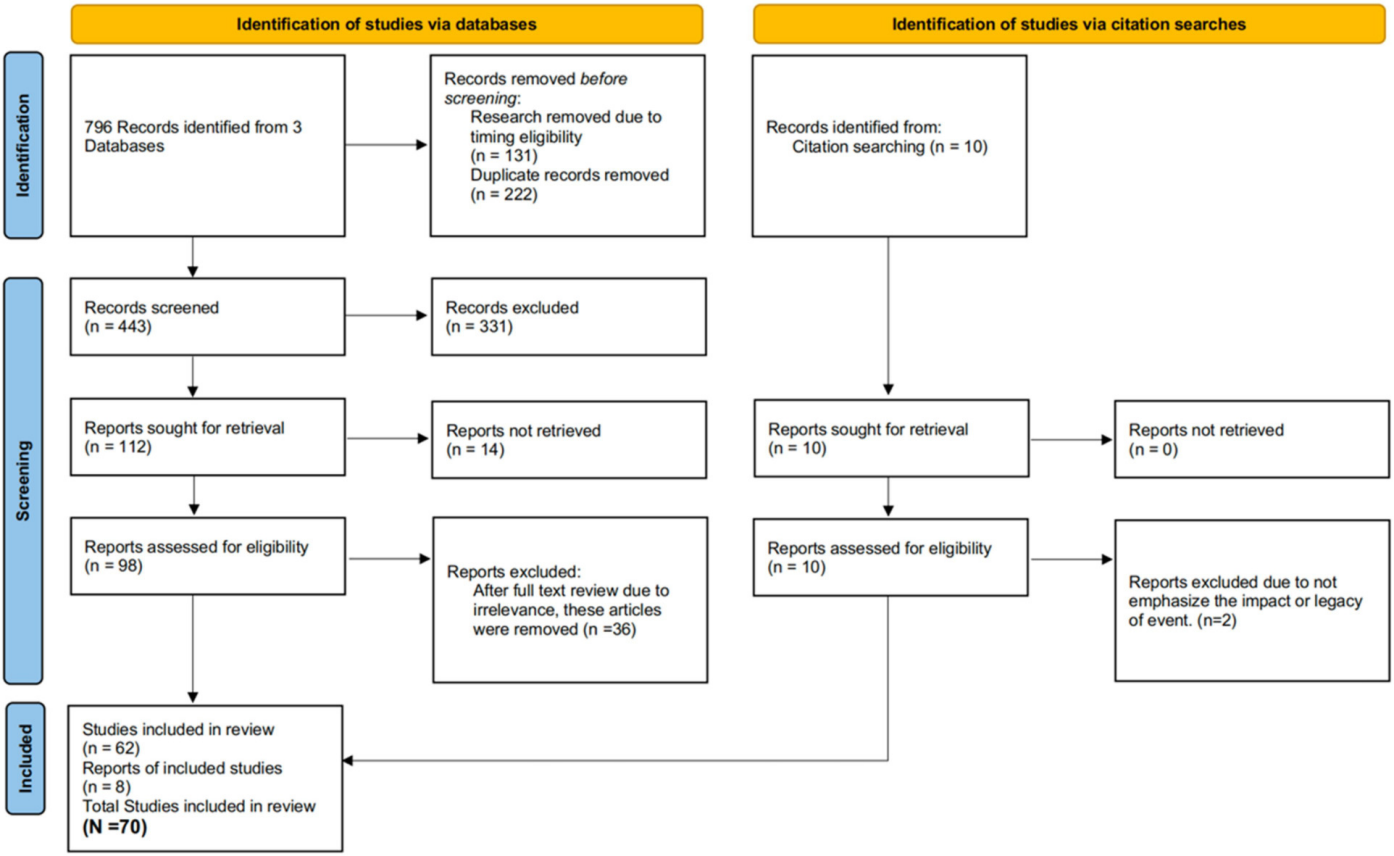
PRISMA—SCR flow diagram of search and eligibility criteria.

Following initial screening, 331 articles were excluded, leaving 112 for full-text review. Fourteen inaccessible articles were removed, reducing the pool to 98. After excluding 36 articles for irrelevance to quality of life, 62 remained. A manual citation search identified 8 additional articles, resulting in 70 total articles included for review. The selection process is summarized in Figure 1.

### Data charting process

The data extraction process from the selected articles was conducted with attention to detail to ensure the collection of relevant information for subsequent analysis. Data extraction employed a standardized form across five domains, event size, type of event, studied population, study design, method and measurement. This structured approach captured demographics, methodologies, and theoretical distinctions (e.g., impact vs. legacy), aligning with the review's objectives and enhancing reliability.

**Figure 2:**
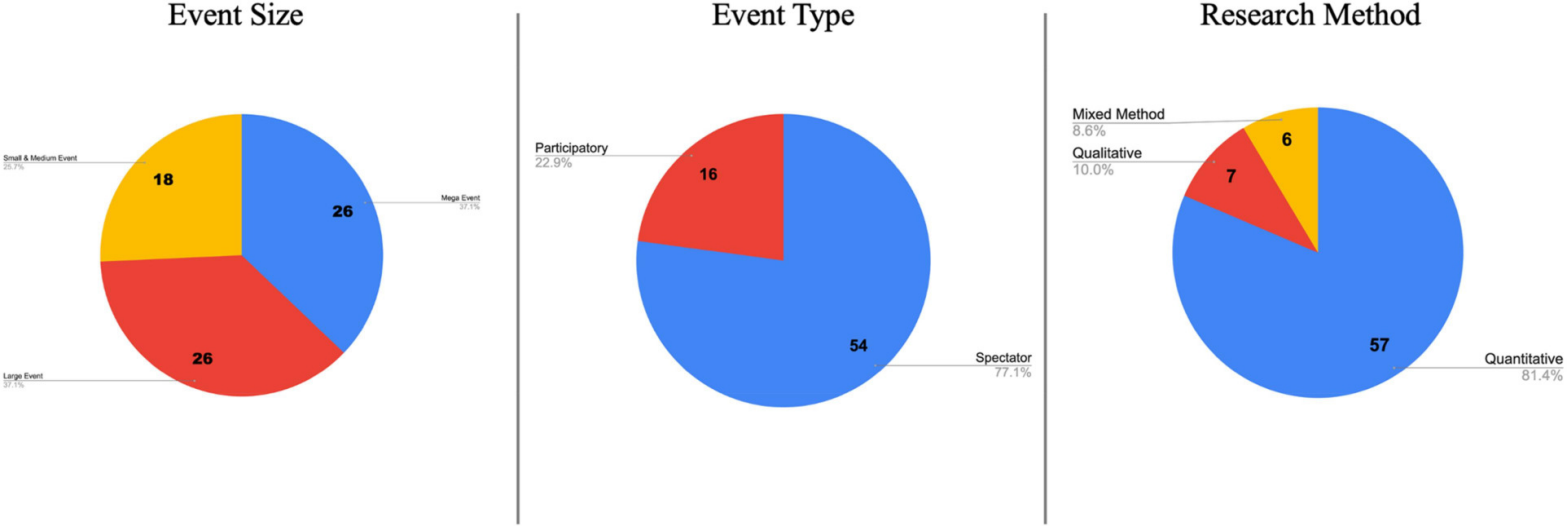
Research trends by event size, event type and research method.

Event typologies as defined by Müller (48) and Gammon (49) were employed to categorize sport events by size: mega events like the Olympic Games and FIFA World Cup, larger international championships, and smaller-scale events such as community sports and national competitions (Figure 2).

## Results & discussion

This section corresponds to the fifth step outlined in the framework proposed by Arksey and O'Malley (1), which involves the reporting and summarizing of the findings. This section addresses the research questions by elucidating the current state of literature in terms of studied context, research designs, theoretical underpins, conceptual distinctions employed across the selected articles. Additionally, the future research directions regarding the impacts and legacies of sport events on quality of life outcomes are also discussed.

### Study context

#### Underexplored potential of small and medium-sized sport events

The existing body of research exhibits a bias towards mega and large-scale sport events, lack of studies on small and medium-sized events. The majority of studies encompassed in this scoping review primarily concentrate on larger-scale sport events. Specifically, mega events account for 37% (*n* = 26) of the research, while large events (e.g., Copa de America, Grand Prix, Asian Games) constitute 37% (*n* = 26), collectively representing over 70% of the studies. Conversely, smaller sport events have received less research attention, with 26% (*n* = 18) of the studies focusing on investigating their impact on the quality of life. This discrepancy underscores the need for more targeted research concerning smaller events. Research on the impacts of the Olympic Games predominates within the mega events category, with these games being the subject of 15 studies, accounting for 22% of this category. The FIFA World Cup follows as the second most studied event, featured in seven analyses.

This imbalance not only highlights a clear research gap, but it also distorts our understanding of quality of life by privileging mega-event contexts. Many of the positive outcomes identified in studies of mega events may be influenced by the substantial impacts inherent to such large-scale spectacles, yielding favorable results that are nevertheless limited and potentially reflective of a temporary “feel-good” effect (15). Furthermore, it reinforces the assumption that improvements in quality of life can only be achieved through large-scale events, suggesting that these spectacles alone are capable of delivering holistic impacts to host city residents and that quality of life is inherently tied to large exogenous shock. This approach captures tangible and highly visible transformations but simultaneously mitigates, the unique and potentially more intimate impacts that small and medium sized events may generate for the individuals and communities involved.

#### Broadening understanding through the impact of different event types

The current literature reveals a limited understanding of how different types of sport events influence on quality of life (e.g., spectating vs. participating, one-off vs. reoccurring). Majority of the studies, representing 77% (*n* = 54), concentrate on spectator oriented events.

Participatory events like marathons, triathlons, and community leagues involve active engagement and may lead to sustained behavioral changes (50, 51), enhanced well-being for both local and non-local participants (52–54), greater community engagement, and volunteerism (7). These effects differ from those seen in spectator events, highlighting the need to distinguish between event types.

Research predominantly examines one-off mega or large-scale events (*N* = 40), which have been associated with positive perceptions of social-psychological, urban, and economic impacts (55, 56). However, such benefits often wane over time (8). For instance, perceived economic gains decline post-event (34), community attachment and perceived infrastructure improvement gradually diminishment after the event concludes (57). This suggests that such events may only temporarily elevate well-being and perceptions feelings (58).

Recurring events, such as annual marathons and seasonal championships, foster stronger community attachment (18), promote personal growth, social bonds (53), and sustained tourism support (59). Their repetitive nature encourages familiarity, long-term engagement, and resident investment (60).

Among reoccurring events, marathons are well researched, with ten studies reflecting their global popularity and a 48.4% increase in participation from 2008 to 2018 (52). Six studies focus on participant well-being (52–54, 61–63), while four examine community-level impacts (10, 12, 18, 64).

#### Demographic insights into quality of life outcomes

A large portion of existing research 78% (*n* = 55)—analyzes the effects of mega sport events and spectator activities on the overall host community demographic level. This general approach lays an essential groundwork for evaluating how sport events affect the perceived quality of life of residents, by tracking changes in their perceptions before and after the event. However, quality of life outcomes may vary depending on different populations studied (10).

Recent research has increasingly examined the impact of major sport events on specific demographic groups, reflecting a shift toward more targeted approaches. Al-Emadi et al. (2) explored the Qatar World Cup's effects on migrant blue-collar workers, offering a unique perspective. Teare et al. (67) studied Canadian youth during the 2010 Olympics, finding increased belonging in venue areas, though this did not lead to higher overall life satisfaction. These findings suggest that mega event impacts are unevenly distributed and highlight the value of segmented analysis for deeper theoretical insights (2, 67).

### Research design

#### Lack of longitudinal research design

The majority of studies (*n* = 43, 61%) used cross-sectional designs, while fewer (*n* = 13, 18%) employed longitudinal methods, including two repeated cross-sectional and two two-wave designs. The reliance on cross-sectional studies limits understanding of long-term and causal effects on host communities (68, 69). Among longitudinal studies, most (6 of 13) had only two time points, pre-and post-event and seven were conducted within a year of the event. While this design captures baseline and immediate changes, the application of multiple time points (i.e., three or more) is vital for accurately discerning the specific event's influence on changes in sports participation (70). Without multiple time points, claims about sustained legacies remain speculative, undermining policy guidance.

#### Heavy reliance on quantitative method

The majority of studies investigating the impact of sport events on quality of life have relied on quantitative methods (*n* = 57), outweighing the use of qualitative (*n* = 7) and mixed methods (*n* = 6) approaches, pointing out a significant gap in the use of qualitative and mixed method design. The multifaceted nature of quality of life outcomes presents a distinct opportunity to integrate qualitative research methods, thereby offering deeper insights and more nuanced support for understanding perceived impacts.

#### Subjective measurements as the predominant measurement

Research has been heavily relying on subjective measurements to determine the perceived impact by the researched audience. 62 studies, 88% of the research utilizes subjective measurements to evaluate quality of life impacts. However, there isn't any consistency among the scales used, different research employed various different measuring tools and applied various scales. The most commonly utilized survey, Kaplanidou's (34) developed survey of quality of life (*n* = 4).

While these subjective measures provide valuable insights into the self-evaluation of the researched audience, quality of life can also be assessed through objective measurements, as highlighted by Cummins (71) and Felce and Perry (72), it is crucial to incorporate both types of measurements when evaluating quality of life.

### Inconsistency in theory application

A key finding from the review of 70 studies is the notable deficiency and inconsistency in the application of theoretical frameworks. Social exchange theory (73) was the most used model, appearing in 13 studies (19%) to explain how quality of life changes affects residents' event support, aiding organizers in understanding these dynamics over time (26, 74). Eight studies adopted a combination of theoretical frameworks. The specific arrangements of theories include social representation theory coupled with social exchange theory (*n* = 4), social exchange theory with the theory of reasoned action (*n* = 1), social exchange theory in conjunction with prospect theory (*n* = 1), social exchange theory with cultural level theory (*n* = 1), and a tripartite framework of activity theory, social emotional selectivity theory, and selection optimization and compensation theory (*n* = 1).

However, most studies (*n* = 37) lacked any theoretical framework. Theories are essential for guiding research design, data collection, and analysis, helping generate testable hypotheses and meaningful conclusions; without them, researchers may struggle to draw meaningful conclusions, limiting the applicability and advancement of knowledge in the field.

### Uncovering consequential and underexplored variables

The research consistently shows a positive link between sport events and quality of life, confirmed by 55 studies. However, the psychological mechanisms underlying this relationship remain underexplored. Of the 70 studies analyzed, only a fraction investigated underlying mechanisms in the relationship between event and quality of life outcomes (*N* = 18). Studies conducted by Bravo et al. (75) and Kinoshita et al. (76) provided insights on the roles of pride or social cohesion, explaining how these psychological factors influence the relationship between perceived sports event impact and quality of life outcomes. This indicates an underexplored area, highlighting the need for future research to delve deeper into the antecedents and consequences of this relationship.

### Recommendations for future research

This section addresses the third and final research question, discussing the recommendations for future research based on the 70 articles reviewed.

#### Event type and population diversification

Research on the impact of small and medium sized events warrants further exploration, as these events can generate similar positive outcomes to larger events, such as pride and collective sense of belonging (10). Additionally, they are capable of creating unique positive outcomes distinct to mega/large events such as, local para-sporting events have been shown to positively impact quality of life and provide educational benefits that foster inclusivity and understanding within society (77). These factors all contribute further understanding of the holistic impact sport events on quality of life outcomes across diverse social groups.

In regard to event types, participatory and recurring events deserve greater scholarly focus due to their unique role in fostering resident attachment (18) and behavioral change (63). Comparative analyses of spectator- vs. participant-oriented events and one-off vs. recurring formats could clarify the conditions under which certain events yield more significant, lasting advantages, or pose greater challenges. Future research should explore diverse event types and sizes, including able-bodied and para-sporting events, and investigate the distinct psychological mechanisms affecting both participants and spectators (52).

A more nuanced understanding of event impacts across diverse social strata is needed. Adopting a meso-level approach, targeting specific communities, social groups, and organizations (78), can address this gap. For example, focusing on marginalized groups such as individuals with disabilities, those from lower-income backgrounds (3, 77) could clarify whether event benefits are equitably distributed (65). Future research should develop frameworks capturing how event legacies vary across social groups (79). These insights can help policymakers and organizers design targeted interventions to ensure major sport event benefits reach often-overlooked populations (2) (Table 1).

**Table 1:** Outcome differences based on event types.

Event type	Studied population	Key outcomes	References
Spectator-Oriented	Spectators, local communities, tourists	Generates positive psychological resources, social capital, pro-social behaviors.	(4) (3)
Participatory	Participants, runners, organizers, veterans with disabilities	Fosters resident attachment, promotes behavioral changes, may act as a positive intervention tools.	(65) (54) (66)
Recurring	Local residents, Participants	Sustained community engagement, event attachment, and the importance of service quality.	(62) (18)
One-Off	Residents	Event impact may be short lived. High visibility and broader resident perceptions underscore the importance of holistic legacy planning to enhance quality of life.	(26) (67)

#### Methodological advancements

Current research lacks longitudinal studies needed to establish causal links and assess long-term effects of sport events. While cross-sectional research has identified critical links between holistic event impacts, legacies, and individual quality of life outcomes (9), they primarily capture short-term outcomes. In studies exploring event's impact and legacy to quality of life, it is crucial to investigate both short-term and enduring outcomes (80). To fully understand both immediate and enduring effects, more longitudinal research extending over longer periods is essential (68).

More application in mixed methods research would strengthen this field of literature, especially qualitative research, would strengthen this field by capturing the depth and contextual nuances of how sport events affect individual and community well-being (81). These insights, derived from diverse perspectives such as in-depth feedback from athletes, residents, and event organizers (65, 82), can provide complementary understandings of the nuanced ways sport events impact quality of life (83). Future research adopting mixed methods could offer a more comprehensive understanding of the relationship between perceived event impact and quality of life, supporting prior calls for such approaches (52, 68) (Table 2).

**Table 2:** Main findings.

Trend	Gap	Future research direction
Research predominantly focuses on larger-scale sport events	Small/Medium sized events	Investigate the impacts of medium and small-sized events, beyond just marathons, to understand their unique contributions.
Emphasis on spectator/one-off events	Participatory/recurring events	Broaden the scope to include diverse event contexts, examine unique impact derived from participatory and recurring events.
Primarily focused on the resident population as a whole	Insufficient comparison of event impacts across different demographics.	Target research to compare impacts on various demographic groups, providing a nuanced understanding.
Heavy reliance on quantitative cross-sectional methods.	Scarcity of longitudinal and qualitative evaluations.	Employ longitudinal studies and mixed method designs to develop a comprehensive view of event impacts and legacies.
Predominantly subjective measurements.	Need for diversified measurement approaches.	Integrate objective measures with subjective scales to enhance the robustness of findings.
Inconsistency in theory application	Theoretical frameworks are often underutilized.	Apply robust theoretical frameworks to elucidate the role of quality of life beyond event support.
Ambiguity between the approach utilized to study “legacy” and “impact”	Lack of clear definitions and measurement guidelines	Clearly define and differentiate legacy and impact studies using precise data collection methods.
Positive relationship established between perceived event impact and quality of life outcomes	Inadequate exploration of the mechanisms in the relationship between event impact and quality of life and various consequences.	Potential in further unpacking of the underlying mechanisms that explains the antecedents and consequences within this relationship.

#### Objective measurement integration

Objective measurements of quality of life encompass various aspects, including economic benefits, health improvements, and social cohesion. These dimensions can be assessed using data on employment rates, healthcare usage, and community involvement statistics, respectively (72). A potentially effective tool to connect objective physiological measurement with quality of life research is through Heart Rate Variability (HRV). The value of HRV lies in two dimensions. First, within the health improvement aspect of quality of life, HRV provides an objective indicator of autonomic responses, allowing researchers to capture how sport event participation or spectatorship may function as an intervention for well-being enhancement (84–86). Second, HRV can support the understanding of the emotional mechanisms linking sport events to quality of life. As positive emotions has been demonstrated of playing a central role in translating collective experiences into improved well-being outcomes (75, 87).

#### Theoretical expansion

Despite the multidimensional nature of quality of life, its theoretical application in research remains limited. Adopting more comprehensive theoretical frameworks could provide greater explanatory depth in the understanding of this field. While social exchange theory focuses on perceived benefits to explain residents' event support, frameworks addressing long-term effects and varying event types remain limited.

Self-Determination Theory (SDT) links event participation to the fulfillment of basic psychological needs, autonomy, competence, and relatedness, highlighting how event-generated conditions can promote sustained engagement and lasting well-being outcomes (88). Through application of SDT, it can deepen the understanding of how different event roles (spectators, athletes, volunteers) satisfy needs differently and thus lead to distinct well-being outcomes (89).

Broaden-and-build theory explains how positive emotions derived from experiences can expand individuals' mindsets and foster broader well-being outcomes (87). Applied to sport events, this theory highlights the emotional dimension of sport events across different types, offering deeper insights into the variety of emotional outcomes and their role in shaping quality of life. Enabling the lens to examine whether cognitive evaluations of event impacts elicit positive emotions that can be broadened into enduring psychological resources or sustained actions. Enriching the understanding of prolonged event impact outcomes.

Furthermore, social anchor theory is capable of offering insights into local or reoccurring sport events, how such events can foster social capital and community cohesion by serving as institutions that promote social connections and shared experiences (90). Such diversified theoretical application can enable further investigation on the underlying factors that enriches the explanations of the established relationship as well as the sequential outcomes. Existing research has yet to sufficiently unpack the mechanisms that generate these effects. Specifically, lack of exploration into what uniquely within the sporting context elicits such elevated sense of well-being and how these mechanisms may differ from other large-scale experiences, such as concerts or festivals. A more granular understanding of these sport-specific drivers is needed to clarify how, and why, sports events offer a distinctive contribution to outcomes.

In addition, active participants, such as athletes or staff, may experience different outcomes than spectators, an area often overlooked. Future studies should consider variations in event types, participant roles, and timing to offer a more nuanced understanding of how events influence quality of life. As demonstrated by previous research, event impacts and legacies are perceived across varying event types, demographic groups, and temporal contexts (79). Incorporating more nuanced factors can help advance both theoretical and practical understanding of how sports events shape individuals' well-being.

## Limitations & conclusion

This scoping review encompasses a number of limitations that are important to consider when interpreting the findings. Consistent with the nature of scoping reviews as outlined by Arksey and O’Malley (1), this review did not include an appraisal of the quality of the included research. As a result, the studies considered may vary widely in methodological rigor, potentially affecting the reliability of the review findings. The review primarily cataloged the types and sizes of sport events and the methods they employed, without delving into the mechanisms or depth of these impacts. Furthermore, the articles reviewed overreliance on subjective measures such as self-reported data introduces risks of recall bias and inconsistency, while the restriction to English-language publications may exclude relevant studies and narrow contextual diversity. Additionally, publication bias may lead to an overrepresentation of positive results, inflating the perceived impacts of sport events.

While sport events are intentionally designed to yield societal benefits, the result of this review highlights the need for more rigorous and balanced research approaches. Future studies should prioritize elucidating the underlying mechanisms driving the relationship between sport events and their impacts, moving beyond descriptive cataloging to offer deeper insights into causal pathways. Moreover, there is a need to develop frameworks and practical solutions that enable event organizers and policymakers to maximize quality of life outcomes across diverse social contexts. By addressing these gaps, future research can support strengthening the understanding and implications of sport event legacies and societal value.
